# Exploration of transfer learning techniques for the prediction of PM_10_

**DOI:** 10.1038/s41598-025-86550-6

**Published:** 2025-01-23

**Authors:** Michael Poelzl, Roman Kern, Simonas Kecorius, Mario Lovrić

**Affiliations:** 1 Institute of Interactive Systems and Data Science, Graz University of Technology, 8010 Graz, Austria; 2 Know Center Research GmbH, Sandgasse 34, 8010 Graz, Austria; 3 Institute of Epidemiology, Helmholtz Zentrum München - German Research Center for Environmental Health, 85764 Neuherberg, Germany; 4 Institute for Anthropological Research, 10000 Zagreb, Croatia; 5 The Lisbon Council, Brussels, Belgium

**Keywords:** Environmental impact, Computer science

## Abstract

Modelling of pollutants provides valuable insights into air quality dynamics, aiding exposure assessment where direct measurements are not viable. Machine learning (ML) models can be employed to explore such dynamics, including the prediction of air pollution concentrations, yet demanding extensive training data. To address this, techniques like transfer learning (TL) leverage knowledge from a model trained on a rich dataset to enhance one trained on a sparse dataset, provided there are similarities in data distribution. In our experimental setup, we utilize meteorological and pollutant data from multiple governmental air quality measurement stations in Graz, Austria, supplemented by data from one station in Zagreb, Croatia to simulate data scarcity. Common ML models such as Random Forests, Multilayer Perceptrons, Long-Short-Term Memory, and Convolutional Neural Networks are explored to predict particulate matter in both cities. Our detailed analysis of PM_10_ suggests that similarities between the cities and the meteorological features exist and can be further exploited. Hence, TL appears to offer a viable approach to enhance PM_10_ predictions for the Zagreb station, despite the challenges posed by data scarcity. Our results demonstrate the feasibility of different TL techniques to improve particulate matter prediction on transferring a ML model trained from all stations of Graz and transferred to Zagreb. Through our investigation, we discovered that selectively choosing time spans based on seasonal patterns not only aids in reducing the amount of data needed for successful TL but also significantly improves prediction performance. Specifically, training a Random Forest model using data from all measurement stations in Graz and transferring it with only 20% of the labelled data from Zagreb resulted in a 22% enhancement compared to directly testing the trained model on Zagreb.

## Introduction

The application of machine learning (ML) to predict future air pollution levels or occurrences of high pollution episodes has gained significant traction^1–4^. This growing use of ML in air pollution prediction can be attributed to various factors. Firstly, ML is adept at handling the intricate and often non-linear associations between numerous variables and air pollutant concentrations. ML algorithms are capable of processing large volumes of diverse and complex data, such as atmospheric and meteorological variables. They can uncover complex patterns and relationships that may have a bearing on air pollution levels, which enhances the depth and accuracy of their predictions^5,6^. Secondly, compared to traditional statistical methods, ML models can offer more precise predictions, especially when dealing with vast, high-dimensional datasets^7^. Thirdly, once trained, these models can deliver real-time or near-real-time predictions. Fourthly, ML can be automated and scaled, allowing extensive geographical coverage and continuous updates as new data emerges.

Utilizing ML, researchers can construct predictive models of exceptional accuracy that incorporate a multitude of elements including sources of emissions, meteorological conditions, and geographical attributes^8–10^. This leads to more accurate and dependable air quality predictions^11^. Furthermore, ML methodologies can evolve and learn from additionally collected data and new data sources once they become available, which allows for continual enhancement of their predictive precision over time. Methods for forecasting and predictions include traditional ML-based approaches such as Random Forests^12,13^ and statistical approaches such as autoregressive methods^14^, and deep learning methods^15,16^. In a forecasting task, the estimation of forthcoming pollutant levels is accomplished by making use of past data and usually environmental variables, essentially extending a time series into the future^10,17^. Alternatively, in a prediction framework, the aim is to predict pollutant levels based on measurements from other sources or locations, without taking into account trends of the target pollutant. This is analogous to estimating pollutant levels in areas where measurements are nonexistent or unachievable. Moreover, this approach offers valuable insights into the primary factors influencing specific pollutants.

Despite the advantages of ML techniques, building predictive models is data intensive and requires time and sometimes computationally intensive training. Additionally, those models are domain-dependent, which means, that they need to be trained on a specific problem, e.g. certain measurement stations and/or pollutants. To overcome those restrictions, transfer learning (TL) became popular among scientists^10,18–20^. The intuition behind transfer learning is to apply the knowledge gained in solving one known problem (source domain) to another, related problem (target domain). Although applied in many use cases in various domains such as transferring the knowledge gained from wind park sensors to newly installed ones to avoid training from scratch and to overcome data scarcity, it is hardly used in the field of atmospheric research^18,21^. Hence, it is of particular interest in this work to explore and showcase the utilization of TL in the application domain of predicting air quality.

Predicting the concentration of pollutants for the next day or week is important when, for example, planning outdoor activities. Ma et al.^9,18^ implemented a framework using Bidirectional Long Short-Term Memory (BLSTM) models to forecast PM_2.5_. In the paper from 2019, the authors transferred the knowledge acquired from models trained with small temporal resolution pollutants data to larger temporal resolution. As an outcome, the authors showed that TL can also improve pollution concentration forecasting accuracy in different temporal resolutions compared to directly training a model with data of higher temporal resolution. Dhole et al.^22^ used meteorological data and pollutants to predict PM_2.5_ concentrations. The authors proposed a multi-source knowledge transfer by creating 10 individual source models (one model per measurement station trained with their individual source data) and transferring the knowledge to the target station to predict PM_2.5_. Different ensemble architectures based on CNNs such as CNN-Long Short-Term Memory (CNN-LSTM) and CNN-Gated Recurrent Units (CNN-GRU) were used as source models. The authors showed, that a cumulative prediction with knowledge of each station performs better than using only using the knowledge from a single retrained station. By implementing various retraining strategies, an improvement of 35% compared to directly training the model with limited data of the target domain. In contrast to our study, Dhole et al.^22^ employed a more extensive dataset, comprising 10 source stations, each with 35,000 samples per station, while our dataset consists of 5 stations with 2885 samples per station. Furthermore, their methodology centred on generating hourly forecasts, diverging from our focus on long-term predictions. Additionally, they integrated past pollutant values into their model, whereas our study solely concentrates on predictive modelling, omitting the utilization of historical pollutant data due to its assumed unavailability or limited accessibility. Lastly, while the authors focused on transferring data solely within one city, our study extends this scope by examining the transferability of models between different cities. In the investigation conducted by Cheng et al.^20^, the focus was on knowledge transfer across ten measurement stations to evaluate its transferability. This transferred knowledge was subsequently employed to judiciously choose a suitable source station for training a ResNet-LSTM model. The key aim was to identify a source station exhibiting minimal errors in predicting PM_2.5_ concentrations in different domains.

To forecast air pollutants, conventional statistical techniques such as Auto-Regressive Integrated Moving Average (ARIMA) can be applied. In recent years, contemporary approaches like ML and particularly deep learning (DL) have exhibited superior performance in multiple scenarios compared to traditional methods^17^. Nonetheless, this improvement comes at the cost of increased complexity in model development and longer execution times^23,24^. Grivas and Chaloulakou^25^ utilized Multilayer Perceptrons (MLP) trained on meteorological and time-scale data to forecast hourly PM_10_ concentrations at four stations within the Greater Athens Area. Their developed models outperformed multiple linear regression models, emphasizing the intricate connection between meteorological factors and PM_10_ concentrations. Cai et al.^26^ employed neural networks to predict CO, NO_2_, PM_10_, and O_3_ concentrations along the roadside in Guangzhou, China. Besides showcasing superior accuracy over statistical models, the proposed models also displayed enhanced transferability, enabling predictions for nearby stations. Bekkar et al.^8^ assessed the performance of various deep learning architectures, including LSTM, Bi-LSTM, GRU, Bi-GRU, CNN, and a hybrid CNN-LSTM. Their study, using historical PM_2.5_ and meteorological features of diverse temporal resolutions, revealed that CNN-LSTM surpassed other models, primarily due to its internal architecture’s capability to extract both temporal and spatial features.

The aim of this study is to (1) understand the key features needed to make PM_10_ predictions, (2) to investigate whether the collective knowledge gained by creating a model out of multiple measurement stations leads to a better predictive PM_10_ accuracy compared to choosing a single station, (3) to explore the feasibility of TL, aiming to generalize models trained in one city to another, thus providing insights into potential applications of TL in similar scenarios, and (4) to estimate the number of labelled target samples needed by the transfer algorithm to yield a notable enhancement in performance compared to out-of-domain generalization.

## Materials and methods

### Air pollutant measurements in Graz and Zagreb

**Fig. 1:**
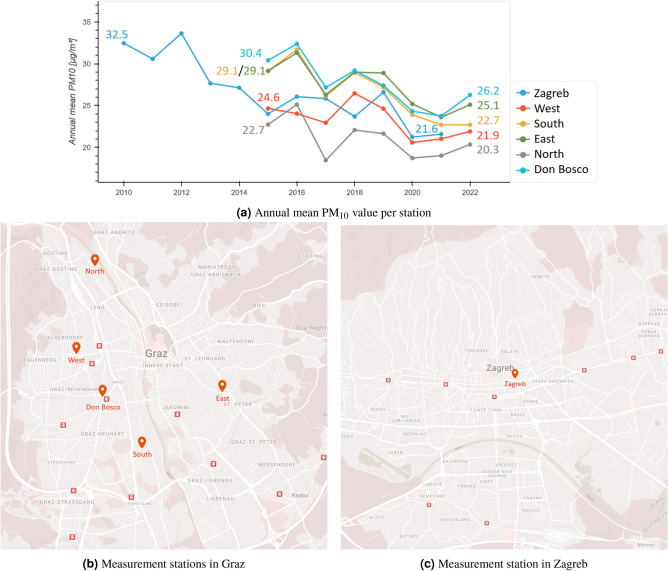
Measurement station location. (**a**) Shows the evolution of the average annual PM_10_ value per station. (**b**) Shows the location of the measurement stations in Graz Don Bosco: $47.055617^\circ$ N, $15.416539^\circ$ E; North: $47.09437^\circ$ N, $15.415122^\circ$ E; East: $47.059530^\circ$ N, $15.466634^\circ$ E; South: $47.041692^\circ$ N, $15.433078^\circ$ E; West: $47.069506^\circ$ N, $15.403728^\circ$ E and (c) Zagreb with coordinates: $45.811389^\circ$ N,$15.989167^\circ$ E. Photos taken from Google maps ®.

Graz is located in the south of Austria and is the second largest city in the country with 298,512 (2023) inhabitants^27^. Zagreb is the capital of Croatia and is located in the north with 768,054 (2021) inhabitants^28^. Graz hosts five governmental measurement stations, namely *Don Bosco* (D), *North* (N), *East* (E), *South* (S), and *West* (W), while *Zagreb* (Z) accommodates three governmental measurement stations, with one having long-term data and considered in this analysis^2^. The selection of stations as research subjects is grounded in the utilization of publicly accessible data^29,30^. A detailed description of the measurement stations in Graz can be viewed in Moser et al.^31^ and for Zagreb in Šimić et al.^6^. The stations in Graz recorded data in the period from 1.1.2014 to 25.11.2021 and the one selected station in Zagreb in the period from 1.1.2009 to 31.12.2020. The data from Zagreb can be accessed via Šimić et al.^29^ and Graz via Lovrić et al.^30^. All recorded measurements are daily averages (24 hours). This results in 2885 samples per Graz station (14,425 in total, without taking missing values into account), and in Zagreb: 4,382 samples. The annual mean PM_10_ evolution per station is shown in Fig. 1a. The geographical locations of each measurement station in Graz are depicted in Fig. 1b, and those in Zagreb are shown in Fig. 1c. Graz is located on the foothills of the Alps and Zagreb is on the slopes of the Medvednica Mountain. The cities exhibit a common characteristic, they occasionally exceed the EU regulation’s^32^ maximum number of days (35) on which a daily mean value for PM_10_ of 50 μg/m^3^ is exceeded. In both cities, the annual mean value for PM_10_ does not exceed the threshold of 40 μg/m^3^, as mandated by the second EU regulation on particulate matter.

### Feature engineering

Data pre-processing, specifically the exclusion of above-average PM_10_ values attributed to specific events like New Year’s fireworks or Sahara dust storms, is conducted following a methodology akin to that outlined in Lovrić et al.^1^.

#### Missing values

The presence of missing values poses a challenge in machine learning, as numerous algorithms are unable to accommodate them. Therefore, it is imperative to employ techniques for accurately detecting and managing missing values, such as by omitting them when necessary. A single sensor measurement comprises multiple features (e.g. temperature, wind speed, etc.). The number of missing values per feature is shown in Table 1. An extended version of this table including various other gaseous pollutants can be found in the supplementary material table *Extended feature per station summary*. The total number of missing meteorological values is 7771, while the total number of missing values for pollutants is 139. Station East has the most missing values. Some features are assumed to be the same in the other surrounding stations, such as humidity, air temperature, and air pressure. These missing features are imputed for station East with the values from the nearest station South. Features such as wind speed or wind peak are considered local and therefore cannot be imputed. These features are discarded. Additionally, the feature radiation of station North is also omitted, since it only occurs in this station and contains many missing values.

**Table 1:** This table summarizes the daily mean meteorological and the air quality feature PM_10_ available for each station.

Feature	Graz Don Bosco	Graz North	Graz East	Graz South	Graz West	Zagreb
$\text {PM}_{10}$ [μg/$\text {m}^3$]	$\checkmark$ (2)	$\checkmark$ (7)	$\checkmark$ (5)	$\checkmark$ (24)	$\checkmark$ (11)	$\checkmark$
Air temperature [$^\circ$C]	$\checkmark$ (4)	$\checkmark$ (10)	$\checkmark$ (1233)	$\checkmark$ (39)	$\checkmark$ (2)	$\checkmark$
% RH	$\checkmark$ (4)	$\checkmark$ (10)	$\checkmark$ (1233)	$\checkmark$ (17)	$\checkmark$ (2)	$\checkmark$
Wind speed [m/s]	–	$\checkmark$ (10)	$\checkmark$ (1233)	$\checkmark$ (4)	$\checkmark$ (2)	$\checkmark$
Wind peak [m/s]	-	$\checkmark$ (10)	$\checkmark$ (1233)	$\checkmark$ (4)	$\checkmark$ (2)	$\checkmark$
Wind direction [Degree]	–	$\checkmark$ (10)	$\checkmark$ (1233)	$\checkmark$ (4)	$\checkmark$ (2)	$\checkmark$
Air pressure [mbar]	–	$\checkmark$ (10)	$\checkmark$ (1233)	–	–	$\checkmark$
Precipitation [$\text {l/m}^2$]	–	$\checkmark$ (48)	–	–	–	$\checkmark$
Radiation [$\text {W/m}^2$]	–	$\checkmark$ (179)	–	–	–	–
$\sum$ Features	3	9	7	6	6	8

The number of missing values is denoted in brackets.

#### Encoding features

The features are coded in a way similar to the approach presented in Bekkar et al.^8^. All continuous wind degree values are expressed as one of multiple classes. 16 classes are used, resulting in $22.5^\circ$ per class. For instance, the wind direction of $8^\circ$ is labelled as class *N* (from $348.75^\circ$ to $11.25^\circ$) and the wind direction of $210^\circ$ is expressed as class *SSW* (from $191.25^\circ$ to $213.75^\circ$). This transformation is done because it reduces variability in wind direction. For machine learning, each category is later changed into an ordinal feature, since most models can only handle numeric values.

#### Temporal features

Apart from the features present in the dataset from the measurement stations, additional temporal features may better explain the concentration of PM_10_, as previously observed in Lovrić et al.^1^. These features are considered global on a city level, as they affect each station in one city. Additional temporal features used in this work are: *dayOfYear* (adds information about the current day [from 1 to 365 or 366]; it is thought to explain much of the seasonal variation in PM_10_ concentration values), *holiday* (adds binary information about whether there is a holiday or not), *dayBeforeHoliday* and *dayAfterHoliday* (indicate one day before and after a holiday; it is assumed, that most polluting travel activities are carried out before and after a holiday), and *weekend* (denotes the binary weekend feature added on Saturdays and Sundays). It is important to note that while features like *weekend* and *dayOfYear* maintain consistent meanings across all stations in both cities, *holiday*, *dayBeforeHoliday*, and *dayAfterHoliday* may encode different semantics due to differences in holiday schedules between the cities.

### Data analysis

When preparing data for ML models, it is crucial to investigate whether certain features, including both those inherent in the dataset and engineered ones like temporal features, contribute significantly to predicting PM_10_ concentrations. This analysis helps determine if these features can explain variations in PM_10_ values to a certain extent. A widely used method for assessing feature importance, particularly for non-linear data, involves calculating the mean decrease in impurity across all decision trees within a Random Forest (RF)^33^. Therefore, a RF is trained to predict PM_10_ concentration which is later used to outline the impact of temporal and meteorological features that explain PM_10_ concentrations. Expanding upon the previously discussed features, we introduce an additional temporal feature known as $PM_{10}$*-lag* into our analysis. Subsequently, we thoroughly examine its influence on the model’s predictive performance. Lag values are adept at capturing dependencies within time series data, making them particularly valuable in time series analysis^6,34,35^. In addition to RF, we applied the Shapley value method to further investigate feature importance. Rooted in game theory, this method has become a widely used approach for analyzing the contributions of individual features in various machine learning models^36–38^. The Shapley value method provides insights into how much each feature positively or negatively contributes to the model’s prediction- in our case, the PM_10_ concentration.

### Model training and algorithms

For air pollution concentration modelling, several approaches were used: (1) a Random Forests regression (RF)^12^ based on our previous studies^1,6^; (2) Prophet (PRH)^23^ and (3) four deep learning architectures, namely a Multilayer Perceptron (MLP)^39^, (4) a Long Short-term Memory Network (LSTM)^40^ network with one LSTM and a 1-dimensional Convolutional Neural Network (CNN)^41^ and finally (5) Neural Basis Expansion Analysis for Time Series (N-BEATS) which have outperformed many models in various ML competitions^42^. The predicted variables (target or outcomes) in this study are the pollutant concentrations at a daily average frequency (PM_10_) at all the locations wherever measured, while the independent (input) features are the temporal and meteorological variables. The models operate under the assumption that the levels of PM_10_ and gaseous pollutants can be predicted using temporal and meteorological variables treated as separate and independent factors. Training data (source domain) consists of all available data from one measurement station (station-level) or from the concatenation of two or more measurement stations of Graz (city-level) and the test set (target domain) stems from all data of another station in the target domain which can either be station Zagreb or one station in Graz. Since the domain of the training data is different from that of the test data, this is considered an out-of-domain generalization (OODG). The intuition behind that is to test the predictive performance of a model tested on unseen data. The experiments are separated into station-level and city-level out-of-domain generalizations. Station-level OODG, uses the data from one station to train a model and tests it on the target domain Zagreb, whereas city-level OODG, uses the data from various stations to train the model. OODG in this work serves as a baseline to discern performance enhancements achieved through TL. An illustration of station and city-level OODG can be found in the supplementary materials (Fig. 1a,b).

### Transfer learning

In classical machine learning, models are typically trained for specific tasks assuming that training and test data come from the same distribution. However, building individual models for each task can be resource-intensive in terms of computation, time, and expertise. Transfer learning (TL) addresses this by transferring knowledge from a source model to a target model, reducing computational costs and leveraging similarities between domains^43,44^. For example, predicting PM_10_ concentrations in Graz and Zagreb entails the same objective of predicting PM_10_ concentration but varies in the domain (i.e., the city). TL allows passing knowledge from a data-rich source model trained in one city to a target model in another city lacking training data, thus improving performance on the target task. Similarly to the experiments conducted in OODG, our experiments in TL are categorized into station and city-level. For further insights into the transfer learning algorithms, approaches utilized and an illustration of station and city-level transfer (Fig. 1c,d), please consult the supplementary materials, specifically the chapters on *Transfer learning algorithms*, *Transfer learning approaches*.

#### Sample injection

**Figure 2:**
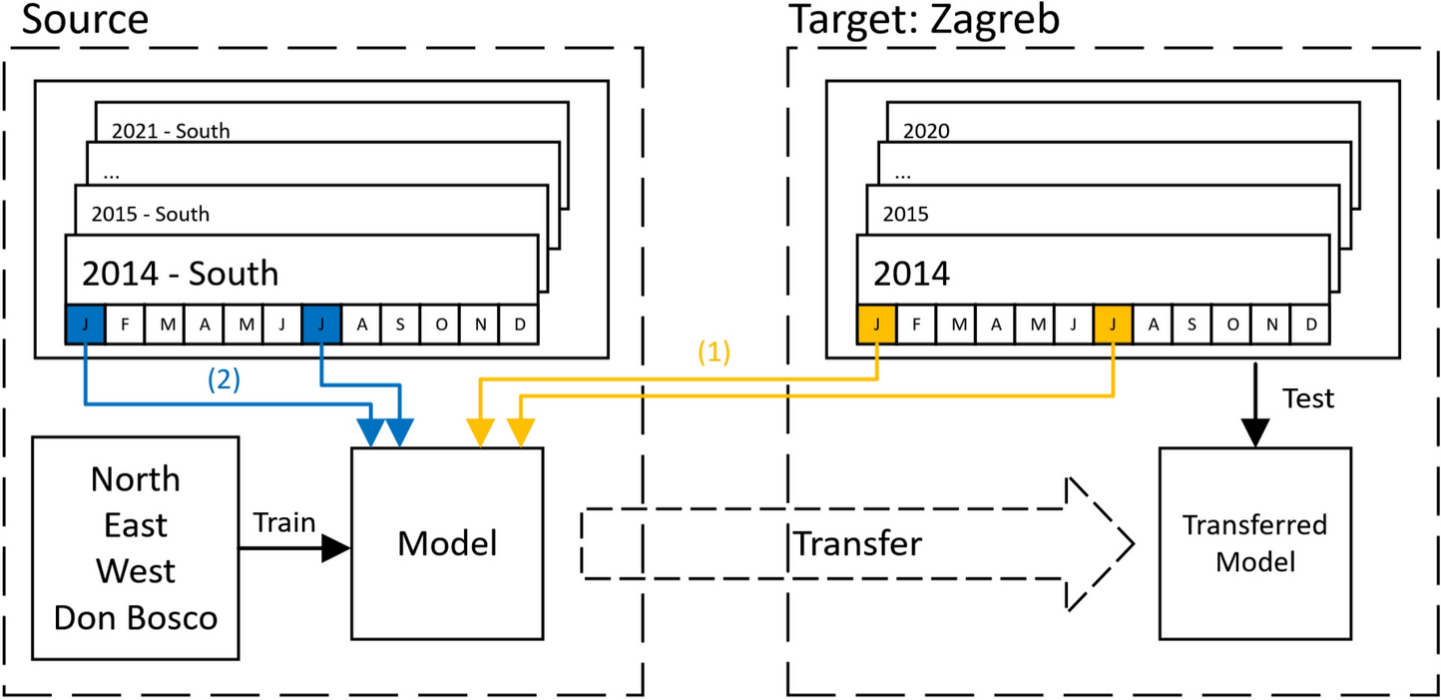
Supervised (1) and unsupervised (2) domain adaptation. Image adapted from Poelzl^45^.

The TL technique used in this work, domain adaptation (DA), can be unsupervised, requiring no labelled data from the target domain, or supervised, requiring a few labelled target samples (PM_10_ values). Unsupervised DA can mimic supervised by adding labelled target samples to the source domain. In this context, providing the DA algorithm with data from the target domain or reusing data from the source domain as target data is referred to as *injection*. Different injection strategies, like station-level and city-level transfer, are explored, with data injected monthly (monthly injection) to preserve seasonality. Labelled target data can be gathered by assuming PM_10_ data is partially available in Zagreb (Scenario 1), or by artificially injecting PM_10_ data assuming numerical similarity between PM_10_ peaks and valleys in Graz and Zagreb (Scenario 2). Figure 2 depicts monthly city-level injection for Scenario 1, where labelled target data for January and July are transferred. In Scenario 2, labelled data from the source domain (station Graz South) replaces target data (station Zagreb), making it unsupervised.

### Evaluation

To measure prediction performance, we use the normalized root mean squared error (NRMSE) to address sensitivity to value ranges between different domains. NRMSE, as defined in Eq. 1, normalizes RMSE by the difference between the minimum *min*() and maximum $max()$ *y* values of a test set, ensuring value range independence. Here, $y_i$ and $\hat{y_i}$ represent real and predicted PM_10_ values, respectively, with *i* iterating through the values and *n* denoting the total predictions.

1 $$\begin{aligned} {\text {NRMSE}} = \frac{\sqrt{\frac{\sum _{i=1}^n\left( y_i-\hat{y_i}\right) ^2}{n}}}{max(y_1,\ldots ,y_n) - min(y_1,\ldots ,y_n)} \times 100 \end{aligned}$$

## Results

The results are presented across various subsections. Initially, in Section Results on feature importance, feature importance differences are explored, shedding light on their impact on predicting PM_10_ concentration. Subsequently, five models (RF, MLP, LSTM, CNN) are assessed for predicting PM_10_ values based on meteorological and temporal features. Among these models, the RF model emerges as the most promising, leading to exclusive focus on it for further investigation. Furthermore, a comparative analysis between station-level and city-level OODG approaches is conducted in Section Results on city-level and station-level out-of-domain generalization. Section Results on transfer learning applies diverse transfer algorithms to the identified optimal approach, with Section Results on injection methods further investigating the most effective transfer algorithm to enhance transferability.

### Results on feature importance

**Fig. 3:**
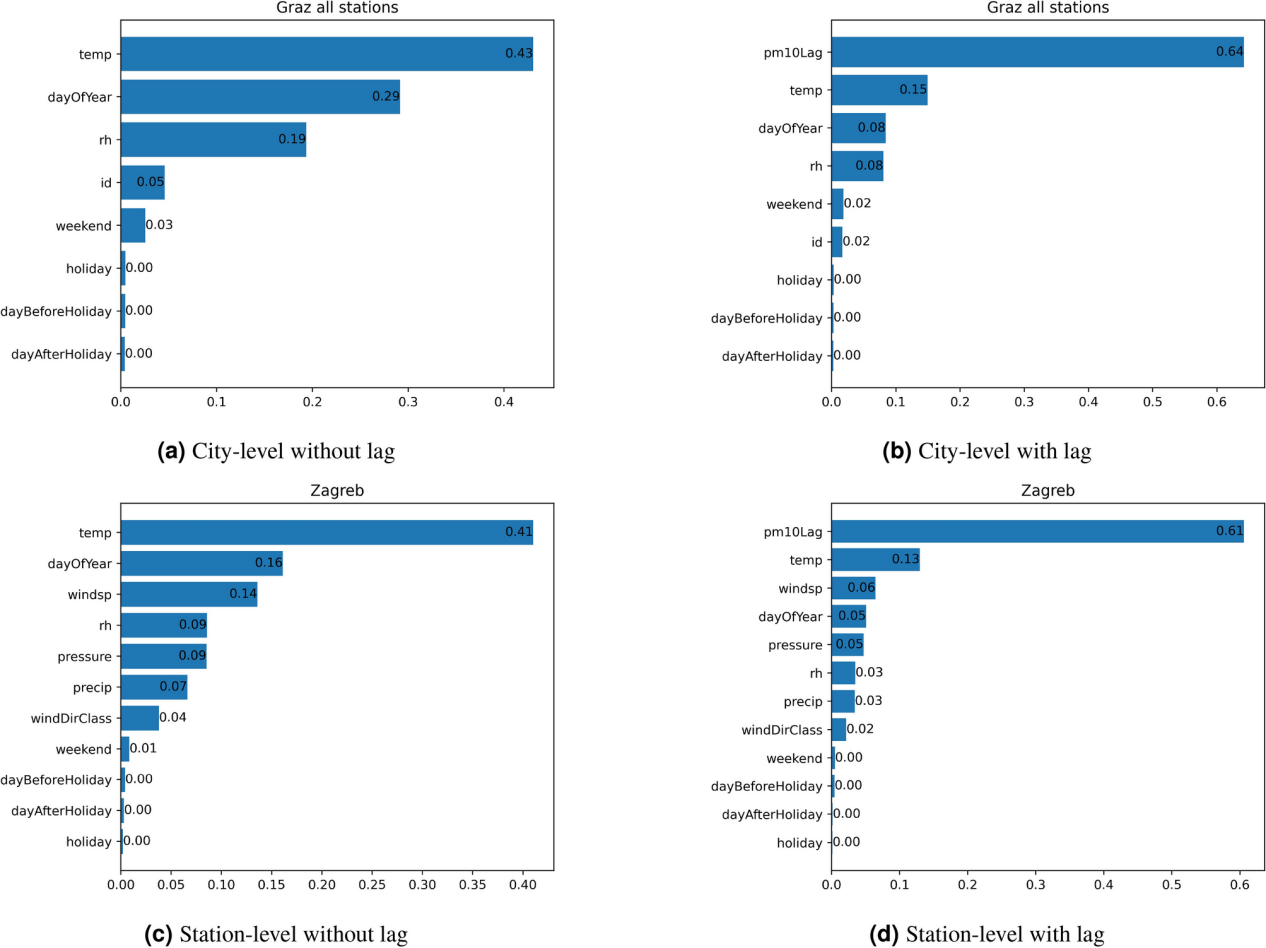
Random forest feature importance. Each subfigure visually represents the significance of individual features, showing the percentage by which each feature explains the PM_10_ concentration. Features include temperature, day of the year, relative humidity (rh), station ID, weekend, holiday, day before and after holiday, wind speed (windsp), wind direction class (windDirClass), precipitation (precip), pressure, and lagged PM_10_ value. (**a**) Depicts the city-level model of Graz without the PM_10_-lag feature, while (**b**) includes this feature. (**c**,**d**) focus on a single station in Zagreb (station-level) using the same approaches. These figures highlight the significant influence of the lagged PM_10_ feature, indicating its explanatory power on the PM_10_ concentration of the subsequent day.

The results from Fig. 3 show that in the city-level model combining features from Graz North, West, South, East, and Don Bosco stations, *temperature* is the most crucial feature (43%), followed by *dayOfYear* (22%) and *relative humidity* (15%). Other features like *weekend*, *holiday*, *dayBeforeHoliday*, and *dayAfterHoliday* are deemed unimportant. The station *id* holds a minor importance (5%). The station *id* encompasses a variety of station-specific properties, including geographical attributes (such as proximity to sources of pollution or surrounding urban infrastructure like tall buildings), which are not accounted for by other features. Adding lagged PM_10_ values to the city-level model elevates this feature to dominance (64%), followed by *temperature* (15%). Similar trends are observed in Zagreb station-level data, where *temperature* is the most important feature, followed by *dayOfYear* and *windspeed*. Feature *windspeed* is absent in the city-level model of Graz as it is not present in every station. When adding the lagged PM_10_ values, this becomes the dominant feature followed by *temperature* and *windspeed*. *dayOfYear* falls behind *windspeed* in this experiment. These findings underscore the importance of meteorological factors like *temperature* and temporal features like *dayOfYear* in PM_10_ concentration prediction, as well as the significance of lagged PM_10_ values in both city and station-level modelling.

**Fig. 4:**
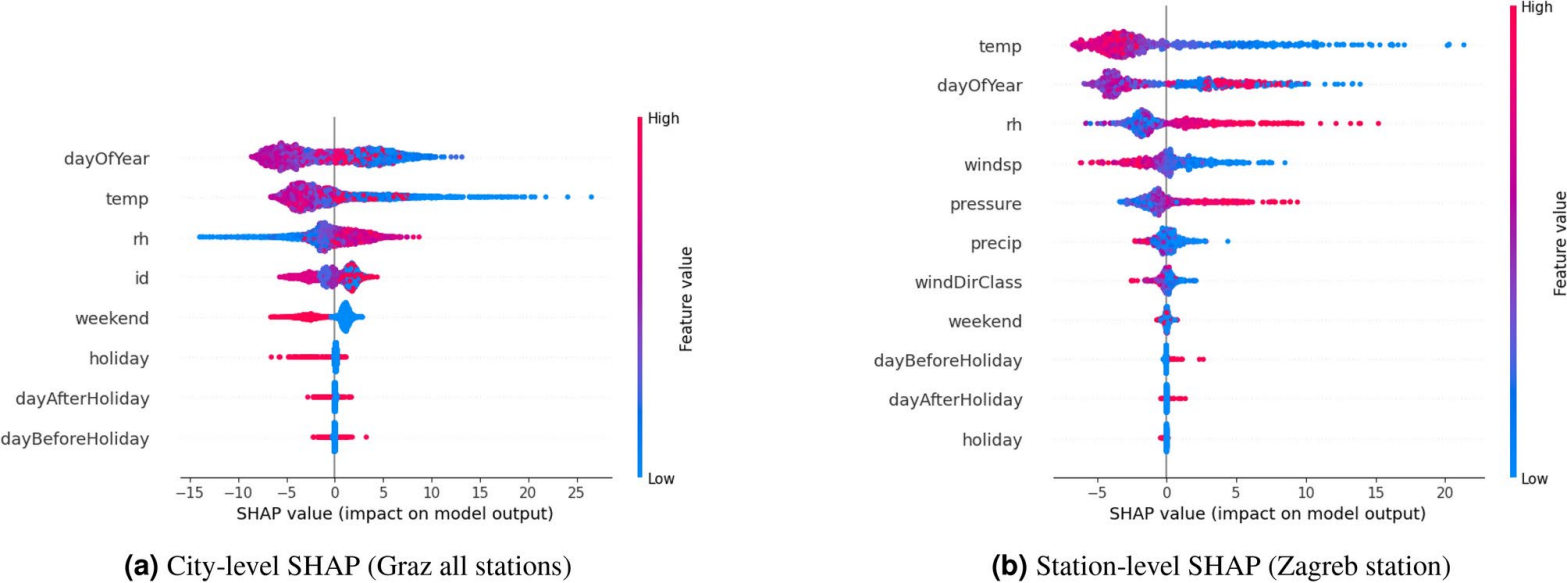
SHAP values. Each subfigure visually represents the SHAP values of individual features, showing their contribution of the predicted pm10. Features include temperature, day of the year, relative humidity (rh), station ID, weekend, holiday, day before and after holiday, wind speed (windsp), wind direction class (windDirClass), precipitation (precip), and pressure. (**a**) Depicts SHAP values of the city-level model of Graz , while (**b**) the SHAP values of the single station in Zagreb (station-level).

To gain a deeper understanding of how features contribute to the model’s predictions, SHAP values are utilized, as shown in Fig. 4. The y-axis ranks the features by their importance, with the most influential at the top, while the x-axis displays the SHAP values, indicating the magnitude and direction of each feature’s impact on PM_10_ predictions. The color scale highlights the feature values, where red represents higher values and blue represents lower ones. In Fig. 4a, *dayOfYear* emerges as the most important feature, showing a seasonal trend: higher *dayOfYear* values, corresponding to summer, have negative SHAP values, likely due to reduced heating activities or increased use of alternative transportation like bicycles, while lower *dayOfYear* values (e.g., winter) exhibit both strongly positive and negative SHAP values, reflecting seasonal variability. One possible reason for the worsening air quality during winter in Graz is its geographical location. Graz is situated in a basin near the Alps, where temperature inversions frequently occur, trapping air pollution^46^. Temperature follows as the second most important feature, where higher temperatures (red points) tend to reduce PM_10_ concentrations, reflected by their negative SHAP values. *Relative* *humidity* shows that lower values (blue) correlate with lower PM_10_ predictions, indicating a distinct pattern. The feature *id* demonstrates variability across stations, as its SHAP values scatter on both the positive and negative sides, suggesting that PM_10_ levels differ notably between measurement stations. Binary features such as *weekend*, *holiday*, *dayBeforeHoliday*, and *dayAfterHoliday* have relatively weaker impacts; however, weekends tend to reduce PM_10_ predictions, likely due to reduced industrial and traffic activity. These results emphasize the significance of temporal and meteorological variability, with *dayOfYear* and *temperature* dominating the predictions while other features provide additional but weaker contributions. The same pattern can be observed for Zagreb as visualized in Fig. 4b. In this case, *temperature* emerges as more important than *dayOfYear*. The feature *windspeed* shows that higher windspeed values contributing negatively to PM_10_ predictions, suggesting that stronger winds help disperse pollutants, leading to lower PM_10_ concentrations. *Pressure* also displays a clear distinction: lower pressure values contribute negatively to PM_10_ predictions, while higher pressure values contribute positively. A similar pattern is observed for *precipitation*, where higher values tend to reduce PM_10_ concentrations. Overall, while the general trends are comparable to those seen in the Graz SHAP values, the relative importance and variability of the features differ slightly, with *temperature* taking precedence in Zagreb. Certain meteorological features, such as *relative humidity*, show distinct differences, with a long tail to the right in Zagreb, whereas in Graz the tail is to the left. These observations highlight subtle differences in feature behavior across the two locations.

### Results on city-level and station-level out-of-domain generalization

**Fig. 5:**
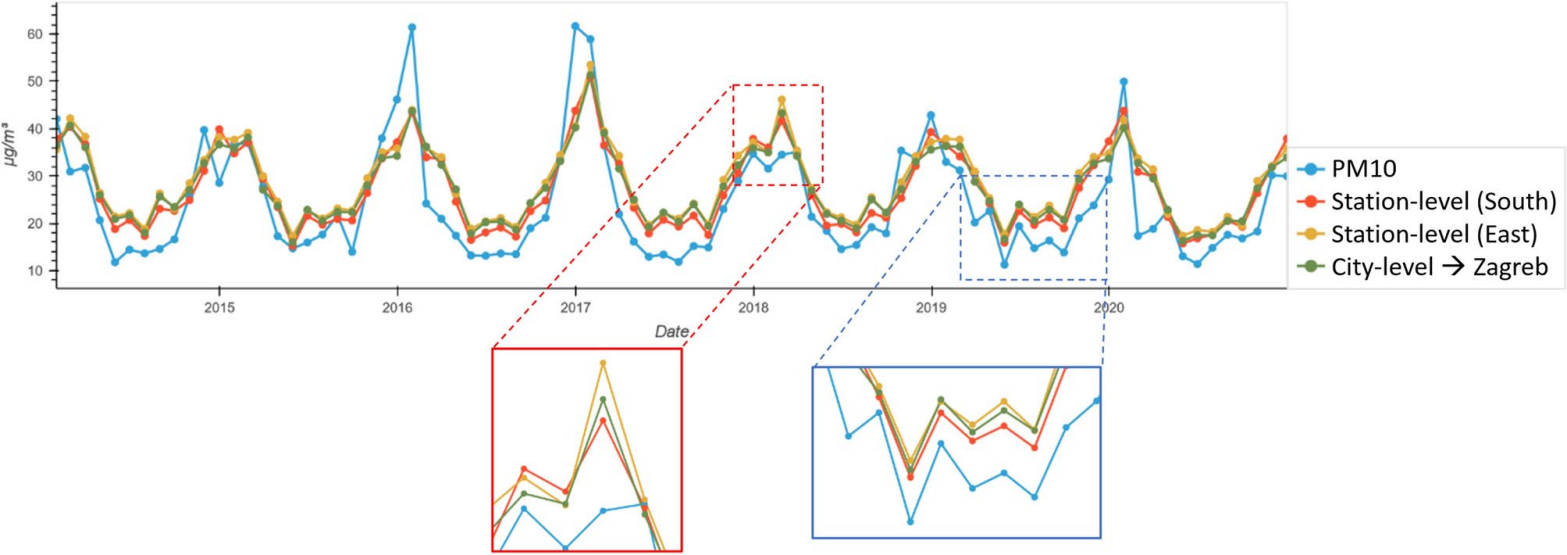
City-level and station-level Random Forest OODG predictions. Shows the difference between the best (station South) and worst station-level (station East) outcome compared to the city-level (all stations combined) approach.

In this experiment, we investigated whether station-level or city-level OODG yields better predictive accuracy. Station-level models trained on single stations in Graz and tested on Zagreb showed significant variation in prediction performance. For example, training with data from Graz Don Bosco resulted in an NRMSE of 9.65, while Graz North yielded an NRMSE of 10.25, indicating inconsistent OODG performance. Conversely, city-level models trained on data from various Graz stations and tested on Zagreb produced predictions falling between the best and worst station-level results. Although city-level models entail longer training times, they leverage collective knowledge from multiple stations. Table 2 illustrates the outcome of this experiment, with Fig. 5 depicting OODG predictions between station and city-level models. The magnified segments in Fig. 5 emphasize prediction variability. Notably, data from station South enhances accuracy during specific periods, while station East slightly underperforms compared to the city-level approach. Crucially, city-level performance consistently falls between the most and least accurate station-level predictions, highlighting the importance of leveraging collective knowledge for improved OODG performance on station Zagreb.

**Table 2:** Station-level and city-level OODG.

	City-level			Station-level				
Stations	N-E-S-W-D	E-S-W-D	N-S-W	N	E	S	W	D
NRMSE	9.32	9.33	9.71	10.26	10.87	9.57	10.24	9.53
Avg. NRMSE	9.44			10.13				

	City-level with PM_10__lag			Station-level with PM_10__lag				
Stations	N-E-S-W-D	E-S-W-D	N-S-W	N	E	S	W	D
NRMSE	7.15	7.13	7.47	8.31	8.01	7.57	8.14	7.39
Avg. NRMSE	7.25			7.88				

The outcomes of city-level RF models, trained on data from various stations in Graz, and station-level RF models, trained on data from a single station in Graz, are presented in the context of experiments conducted on the Zagreb station. Normalized root mean square error (NRMSE) serves as the performance metric. To assess the impact of PM_10__lag features on the prediction performance, the second part of the table shows models including lagged values. In the city-level approach, performance remains relatively stable across directions (N-E-S-W-D and E-S-W-D), with minor distinctions observed, notably, N-S-W performing slightly inferior. Conversely, the station-level approach exhibits higher performance fluctuations; specifically, training on Graz station E and testing on Zagreb yields significantly inferior results compared to training on Graz station S and testing on Zagreb.

### Results on transfer learning

Table 3 provides detailed results for various transfer learning algorithms. We explore unsupervised algortihms Nearest Neighbors Weighting (NNW) , Kullback-Leibler Importance Estimation Procedure (KLIEP), Correlation Alignment (CORAL), and supervised transfer AdaBoost for regression (TrAdaBoostR2), using RF as a regressor. NNW exhibits negative transfer effects, particularly when using data from single stations or a city-level model from Graz, while KLIEP does not yield notable improvements. CORAL displays mixed outcomes, including negative transfer and slight improvements, with a substantial 10% increase when transferring from Graz West to Zagreb. However, overall, it does not consistently enhance predictive accuracy for station Zagreb. In contrast, TrAdaBoostR2 shows significant performance gains of up to 22% when injecting target domain data (413 samples from station Zagreb: months January and July from 2014 to 2020), emerging as the most promising algorithm. Further investigation into its effectiveness at both city and station levels, along with determining the optimal number of target samples required from station Zagreb, is warranted to guide future analysis.

**Table 3:** Transfer Learning algorithms performance comparison.

Source	NRMSE				
	OODG	NNW	KLIEP	CORAL	TrAdaBoostR2
N	10.27	10.06($\uparrow$ 2.1%)	10.2 ($\rightarrow$)	10.97 ($\downarrow$ 6.8%)	8.26 ($\uparrow$ 19.6%)
E	11.05	11.88 ($\downarrow$ 7.5%)	10.96 ($\rightarrow$)	10.55 ($\uparrow$ 4.5%)	9.04 ($\uparrow$ 18.1%)
S	9.57	9.86 ($\downarrow$ 3.0%)	9.56 ($\rightarrow$)	9.040 ($\uparrow$ 5.5%)	7.87 ($\uparrow$ 17.7%)
W	10.26	9.99 ($\uparrow$ 2.5%)	10.26 ($\rightarrow$)	9.22 ($\uparrow$ 10.1%)	7.99 ($\uparrow$ 22.0%)
D	9.64	9.97 ($\downarrow$ 3.5%)	9.64 ($\rightarrow$)	9.19 ($\uparrow$ 4.5%)	7.89 ($\uparrow$ 18.1%)
N-E-S-W-D	9.45	9.68 ($\downarrow$ 2.5%)	9.43 ($\rightarrow$)	9.23 ($\uparrow$ 2.3%)	7.78 ($\uparrow$ 17.5%)

This table shows the NRMSE of various station-level (one letter) and city-level (N-E-S-W-D) RF source models of Graz transferred to station Zagreb. In Supervised TL algorithms TrAdaBoostR2 and NNW we inject 413 (January and July between 2014 and 2020) labelled samples from the target domain (station Zagreb). The performance improvement ($\uparrow$), deterioration ($\downarrow$) and the absence of improvement ($\rightarrow$) achieved with TL compared to OODG is denoted in the braces.

### Results on injection methods

We explored the impact of injection quantity and timeframe on transferability from Graz to Zagreb. The experiments revealed that TrAdaBoostR2’s prediction performance for PM_10_ in Zagreb is highly contingent on injected samples (Table 4). Both the number of injections (from 59 to 821 samples from Zagreb into Graz) and the introduced seasonality pattern (monthly injections in January, February, June, and July) are crucial. This experiment investigates various injection quantities and their associated impact on transferability, distinguishing between the target (Scenario 1) and source injection (Scenario 2), both explained in Subsection Sample injection. While no substantial performance improvement was observed with source injection compared to OODG, our focus remains on target injection. Results of TrAdaBoostR2 with different numbers of injections are presented in Table 4.

**Table 4:** TrAdaBoostR2 results.

Injected Samples	821	413	177	177	117	59	59	
Years	2014, …, 2020	2014, …, 2020	2016, 2017, 2019	2014, 2017, 2019	2014, 2019	2017	2019	
Months	1, 2, 6, 7	1, 7	1, 7	1, 7	1, 7	1, 7	1, 7	

Source	NRMSE							
	OODG							
N	10.27	7.38	8.26	8.81	9.39	9.91	9.30	9.89
E	11.05	7.381	9.04	9.75	11.11	10.59	10.24	9.57
S	9.57	7.369	7.87	8.49	8.59	9.00	9.10	9.29
W	10.26	7.61	7.99	8.53	8.79	9.25	9.39	9.70
D	9.64	7.42	7.89	8.34	9.09	9.32	9.57	9.08
N-E-S-W-D	9.45	7.32	7.78	8.40	9.33	9.58	9.88	8.85

This table shows the NRMSE of various station-level (one letter) and city-level (N-E-S-W-D) RF source models transferred to station Zagreb using the supervised transfer learning algorithm TrAdaBoostR2. Samples are injected from the target station (Zagreb) during the transfer. The number of injected samples depends on the selected years and months. To emphasize the performance improvement achieved by transfer learning, the out-of-domain generalization results in column OODG are added to show the prediction results of each model for the target station Zagreb without transfer.

### Impact of dataset size

To further improve transfer learning, besides model-based improvements such as hyperparameter tuning or transfer learning-based settings (e.g., the number of injected values during transfer), the size of the training set might also influence the accuracy that can be achieved^47^. Therefore, additional data from 2009 to 2013 were retrieved, processed, and added to the original dataset, increasing its size by 60%. Initially, there were 2885 (total 14,425) samples per station; now, instead of 2885, there are 4668 samples (total 23,340). As a transfer learning algorithm, TrAdaBoostR2 was used, and data from January and July of each year were injected, resulting in 708 injected values, which account for approximately 16% of the total available PM10 data from the Zagreb station. The results in Table 5 highlight improvements in both scenarios, OODG and TL. The city-level model transferred to Zagreb showed an improvement from 8.5 to 7.86, representing a 9% increase. This demonstrates that, in this specific application, the number of training samples plays a significant role in enhancing transferability and achieving higher accuracy. However, this improvement in accuracy came at the cost of increased training time, which rose from 1 min and 48 s to 4 min and 17 s. This demonstrates that, in this specific application, the number of training samples plays a significant role in enhancing transferability and achieving higher accuracy, though with a higher computational burden.

**Table 5:** Extended dataset TrAdaBoostR2 results.

Source	OODG		TL	
	2014–2022	2009–2022	2014–2022	2009–2022
	NRMSE			
N	10.225	8.789	8.26	7.9
E	9.5	8.722	9.07	7.93
S	9.06	8.96	7.93	7.47
W	9.873	9.609	8.08	7.67
D	8.776	8.815	8.5	7.95
N-E-S-W-D	8.686	8.64	8.5	7.86

This table presents the NRMSE of various station-level (single letter) and city-level (N-E-S-W-D) RF source models transferred to station Zagreb using the supervised transfer learning algorithm TrAdaBoostR2. Samples from the target station (Zagreb) are injected during the transfer. Two time frames-2014-2022 (14,425 training samples) and 2009–2022 (23,340 training samples)-are compared to illustrate the impact of a larger dataset on both OODG and TL performance.

## Discussion

In this study, we combined data analysis and machine learning to obtain the most information in the field of air pollution investigation. In our feature importance analysis, we highlighted relevant features such as temperature and *dayOfYear* for making PM_10_ predictions. Additionally, we demonstrated the impact of lagged PM_10_ values (in this case, the PM_10_ concentration of the previous day) on PM_10_ predictions. The identified relevant features were further utilized to select a suitable ML model. Among the options explored, including Random Forests, LSTM, NBeats, CNN-LSTM, and MLPs, Random Forests exhibited the best performance in predicting PM_10_ concentrations, particularly in terms of OODG. The rationale behind Random Forests outperforming other architectures such as MLP, CNN-LSTM, or LSTM may be attributed to factors such as the limited availability of training data and the relatively lower complexity of the dataset. In Chae et al.^48^, among other methods, CNN and LSTM were utilized to predict PM_10_ and PM_2.5_ concentrations. In contrast to our investigation, their study employed a dataset exceeding 4 million samples for predictive modelling and increased data complexity by integrating additional air pollutants such as SO_2_, CO, O_3_, and NO_2_ alongside meteorological variables. It is worth noting that NBeats might not have achieved the performance of Random Forest since it is designed for univariate time-series forecasting. However, in our case, we perform multivariate time-series prediction, as we have multiple features to predict PM_10_ concentrations and do not consider past PM_10_ observations^42^.

We demonstrated that in an OODG scenario, the collective knowledge gained by training a model using data from multiple stations in Graz and testing on station Zagreb (average NRMSE 9.52) is, on average, better than training a model based on a single station in Graz and testing it on Zagreb (average NRMSE 10.12). One possible explanation might be the increased training data and higher flexibility of the model as it trains on a more diverse dataset. After establishing the baseline using OODG to make predictions on Zagreb based on a model trained with data from Graz, we successfully applied transfer learning by employing domain adaptation algorithms, both in a supervised (where the Zagreb target station requires to have PM_10_ values) and unsupervised (where the Zagreb target station does not require to have PM_10_ values) manner. The results clearly showed that the supervised domain adaptation algorithm TrAdaBoostR2 can significantly improve the PM_10_ prediction performance (up to 22%) compared to OODG. However, the other unsupervised algorithms used, such as CORAL, KLIEP, and NNW, showed a range of outcomes from minor improvements to slight deterioration compared to TrAdaBoostR2. CORAL showed both performance deterioration and improvements, as can be seen in Table 3. NNW and KLIEP did not demonstrate any noticeable improvement in our experimental results. Although showing performance improvements compared to unsupervised TL algorithms, supervised algorithm TrAdaBoostR2 has some downsides as it requires labelled target data injected during training. Estimating the number of injections is not straightforward; as depicted in Table 4, the number of injections and the years of the injected months influence the performance of the transferred model. There exists no clear pattern determining a “good” year from which data can be injected. It might be due to local weather conditions that differ between the cities, strongly influencing PM_10_ concentration. However, the empirical study revealed that injecting 177 samples (from at least January and July from 3 different years) assures an improvement compared to OODG.

In contrast to our work, transfer learning in the literature studied Deng et al.^19^, Dhole et al.^22^, Ma et al.^9^, Fong et al.^10^, Cheng et al.^20^ is mostly implemented using parameter-based approaches with underlying CNN-LSTM, CNN-GRU, or LSTM models, which typically require a larger number of data samples than our transfer approach. For example, Cheng et al.^20^ successfully implemented CNN-LSTM and demonstrated successful results in hourly predictions with parameter transfer. However, they utilized 10 source stations, each with 35,000 samples per station, giving a total of 350,000 training samples, compared to the 14,000 samples used in our case. Furthermore, the literature predominantly focuses on short-term prediction (hourly/daily/weekly), with the common scenario being data scarcity within a single city. Additionally, most authors concentrate on forecasting, which is a subcategory of prediction, considering past air pollutant concentration values to estimate future behaviour. For instance, in Fong et al.^10^, the approach involves forecasting, as data from the past 6 days are used to predict air pollution concentration for day 7. Our approach stands out as novel in the field of air pollution concentration prediction, demonstrating the effectiveness of training a model in one city and transferring it to another city, even with limited training samples, to make long-term predictions. This underscores how transfer learning can enhance cross-city predictions, even when little labelled air pollution concentration data is available, potentially enabling data-rich cities to improve prediction accuracy in data-poor cities over extended periods, despite differences in air pollution data between cities.

## Conclusions

This study addresses the challenge of data sparsity when predicting air pollution concentration levels based on meteorological and temporal data in cities, impeding precise forecasts. To mitigate these data limitations, we conducted an in-depth exploration of transfer learning (TL) techniques and their feasibility in this application domain. This exploration facilitated the effective transfer of knowledge from the data-rich city of Graz to Zagreb allowing us to make more accurate predictions in Zagreb.

Our analysis employing Random Forests (RF) demonstrates the significant predictive roles of both temporal features specifically, the day of the year and meteorological features in predicting PM_10_ concentrations. Consequently, for the development of machine learning models such as LSTM, CNN, CNN-LSTM, RF, and MLP, only relevant features were selected based on our findings. RF demonstrated superior performance among the models assessed, likely due to its ability to effectively handle a limited amount of training data.

Based on our findings, RFs are further investigated for out-of-domain generalization (OODG). We evaluated the performance of RF models trained at the station level (using data from individual measurement stations in Graz) and at the city level (using data from multiple measurement stations in Graz) when predicting PM_10_ concentrations in Zagreb. Station-level models showed increased variability in their prediction performance across different stations in Graz, leading to inconsistent OODG. In contrast, city-level models demonstrated more consistent predictions, although they required longer training times due to their reliance on data from various stations. These findings suggest that, in this specific scenario, city-level models are preferred over station-level models in predicting values for stations in different domains.

To enhance OODG prediction performance further, we applied the same station and city-level approach with RFs to various unsupervised TL algorithms including Correlation Alignment (CORAL), Kullback-Leibler Importance Estimation Procedure (KLIEP), and Nearest Neighbors Weighting (NNW), which do not rely on labelled data in the target domain (such as PM_10_ values in Zagreb). Additionally, we explored the supervised TL algorithm AdaBoost for regression (TrAdaBoostR2), which requires a certain number of labelled target samples. However, the same consistent pattern as occurring in OODG emerged where city-level models exhibited more consistent performance, prompting their consideration for further analysis. The unsupervised algorithms did not yield significant or consistent performance improvements, the supervised algorithm TrAdaBoostR2 with the underlying city-level model achieved a notable reduction in normalized root mean squared error (NRMSE) to 7.3, representing a 22% improvement compared to OODG with an NRMSE of 9.445. This improvement was achieved by providing the supervised TL algorithm TrAdaBoostR2 with target labels (PM_10_ values from station Zagreb) from January, February, June, and July across all years, resulting in 821 values out of 4382 possible samples. Furthermore, selecting January and July of every second year (226 PM_10_ values) also resulted in a significant performance boost, with an NRMSE of 8.1, representing a 14% improvement compared to OODG with an NRMSE of 9.445.

Our study demonstrates the feasibility of transferring ML models between sites, even when only a portion of the pollutant data at the target site is available to the TL algorithm. This capability holds promise in broader contexts, enabling predictions in scenarios with limited data availability. Such applications are particularly relevant for filling in missing data in epidemiological studies. Nevertheless, it is imperative to acknowledge a limitation of TL in this study: while empirical results revealed that injecting 177 samples (from at least January and July from 3 different years) achieves a performance improvement compared to OODG, it remains a tedious task to select the most suitable months from certain years to achieve significant predictive results. This deviation in performance improvement highlights the complexity and potential unpredictability of supervised TL outcomes in this context. In addition to these limitations, the number of training samples also plays a crucial role in determining the transferability and accuracy of the model in the target domain. This adds another layer of complexity, as both data size and the selection of specific months or years need to be carefully considered to achieve significant predictive outcomes.

As next steps, the dataset could be enriched by incorporating data from multiple measurement stations across Europe using the data retrieval tool presented by He et al.^49^. This would expand the dataset and potentially improve prediction accuracy for Zagreb through TL. By including data from stations across Europe, the transferability of measurement stations between different domains-characterized by variations in environmental conditions, emissions, climate, traffic, or industrial activities-could be further explored. This investigation could provide insights into which domain differences are most relevant for transferring knowledge between cities to predict environmental pollution. These insights could also help identify optimal target samples to enhance supervised TL performance.

## Supplementary Information

Supplementary Information.

## Data Availability

The datasets analysed during the current study are available in the Zenodo repository: Zagreb (https://zenodo.org/records/6390135)^29^ and Graz (https://zenodo.org/records/6812067)^2^. The source code is available in the GitHub repository: https://github.com/mipo17/TLTForPredOfPM10.
