# Supplementary material for: Exploration of transfer learning techniques for the prediction of PM10
Source: Sci Rep. 2025 Jan 23;15:2919. doi: 10.1038/s41598-025-86550-6 (PMC11757726; doi:10.1038/s41598-025-86550-6)
Supplement: Supplementary file 1 — Supplementary Information. [file 41598_2025_86550_MOESM1_ESM.pdf]

Supplementary material

Table 1. Extended feature per station summary. This table summarizes the daily mean meteorological and air quality features available for each station. The number of missing values is denoted in brackets.

Feature	Graz Don Bosco	Graz North	Graz East	Graz South	Graz West	Zagreb
O_3 [μg/m^3]	-	✓(6)	-	✓(24)	-	-
NO [μg/m^3]	✓	✓(2)	✓(2)	✓(3)	✓(13)	-
NO_2 [μg/m^3]	✓	✓(2)	✓(2)	✓(3)	✓(13)	-
NO_x [μg/m^3]	✓	✓(2)	✓(2)	✓(3)	✓(13)	-
PM_1 [μg/m^3]	-	-	-	-	-	✓
$PM_{2.5}$ [μg/m^3]	-	-	-	-	-	✓
PM_{10} [μg/m^3]	✓(2)	✓(7)	✓(5)	✓(24)	✓(11)	✓
Air temperature [°C]	✓(4)	✓(10)	✓(1233)	✓(39)	✓(2)	✓
% RH	✓(4)	✓(10)	✓(1233)	✓(17)	✓(2)	✓
Wind speed [m/s]	-	✓(10)	✓(1233)	✓(4)	✓(2)	✓
Wind peak [m/s]	-	✓(10)	✓(1233)	✓(4)	✓(2)	✓
Wind direction [Degree]	-	✓(10)	✓(1233)	✓(4)	✓(2)	✓
Air pressure [mbar]	-	✓(10)	✓(1233)	-	-	✓
Precipitation [l/m^2]	-	✓(48)	-	-	-	✓
Radiation [W/m^2]	-	✓(179)	-	-	-	-
$\sum$ Features	6	13	10	10	9	10

Transfer learning algorithms

Domain adaptation (DA) is a transfer learning technique in which source and target domains are different, but related, whereas the source task and target task are the same. The domain can differ by feature space and/or data distribution, e.g. two different locations. DA can either be implemented feature-based or instance-based. Additionally, there exist supervised and unsupervised solutions. In supervised solutions, a low amount of labelled data in the target domain is available, whereas in unsupervised no data is available in the target domain. Four DA algorithms are explored in this work: (1) TrAdaBoostR2[2] is an instance transfer algorithm, derived from AdaBoost and specifically tailored for regression tasks. Instance-based DA TrAdaBoostR2 takes the samples from the source domain (e.g. features and corresponding labels from stations of Graz) and samples from the target domain (a predefined number of features and corresponding labels from station Zagreb) as input and combines them into a single dataset. During boosting, it decreases the weight of source samples poorly predicted while it increases the weights of target samples poorly predicted. The weight decrease and increase control the impact in the model training: errors made in predicting the source data, therefore, have less impact than errors made in predicting the target samples. This mechanism allows the algorithm to identify source instances that are similar to the target instances while disregarding those that are dissimilar[2]. (2) CORAL[3] is a feature-based algorithm that aims to align the second-order statistics (or second momentum, the covariance) of source and target data. In other words, it minimizes the domain shift by aligning second-order statistics of source and target data. (3) Nearest Neighbors Weighting (NNW)[4] is another instance transfer algorithm that aims to adjust the weights of source instances according to their proximity to the target dataset's neighbors. This algorithm is typically used for a number of data samples more than 10k. For less than 10k, the (4) Kullback-Leibler Importance Estimation Procedure (KLIEP)[5,6] is the preferred instance transfer algorithm as opposed to NearestNeighborsWeighting. This algorithm aims to correct the difference between source and target distribution by reweighting source samples. The reweighting procedure is designed in a manner that minimizes the Kullback-Leibler divergence between the distributions of the source and target datasets. It is typically used as an unsupervised DA algorithm but can be transformed into a supervised one by adding labelled target samples to the source samples.

Transfer learning approaches

Figure 2 exhibits the most common form of transfer learning when it comes to transferring knowledge in neural networks: *parameter transfer*. It aims to train a model with the data from a source domain. In the second training (aka. fine-tuning or retraining), the weights of a variable number of layers are frozen to store the knowledge from the source domain, only a few layers are trained with data from the target domain. This technique enables faster training and eliminates the need for retraining from scratch. Parameter transfer can be found in computer vision tasks such as object classification. A model is trained to categorize dog breeds in the source domain. The same model is later used in a target domain to classify cat breeds by

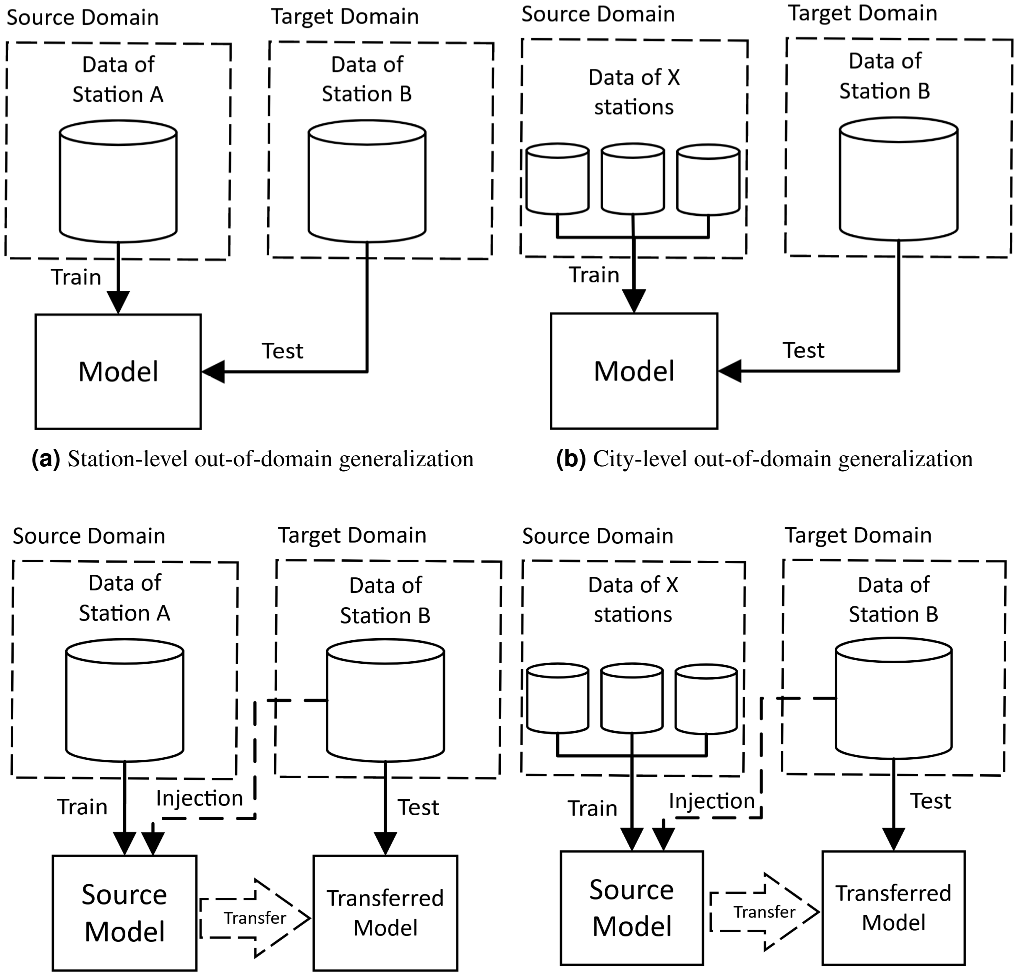

(a) Station-level out-of-domain generalization

(b) City-level out-of-domain generalization

(c) Station-level transfer

(d) City-level transfer

Figure 1. Out-of-domain generalization and transfer learning. Figures (a) and (b) exhibit the difference between station-level and city-level OODG whereas (c) and (d) show supervised station-level and city-level transfer. Image adapted from Poelzl[1]

retraining and fine-tuning. Besides parameter transfer, *instance-transfer* and *feature-transfer* are also present in the literature. In instance-transfer, exhibited in Figure 3, part of the source domain is used together with labelled data from the target domain. The goal is to find the most suitable source data to be reused in the target domain to increase the model performance. This method is mostly used in sample bias scenarios, in which members of a population are more likely to occur in a sample than others. Feature-transfer aims to find ”good” feature representations by continuously learning in the source domain and migrating these into the target domain. In this approach, it is assumed that the domain shift is caused by any data acquisition conditions such as sensor drifts. Moreover, it is commonly used in unsupervised TL tasks, in which no labelled target domain data is available. TL can not only increase performance in the target domain but also decrease it, which is called *negative transfer*[7–9].

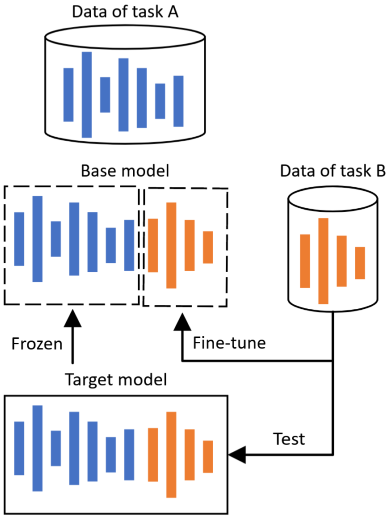

Figure 2. Example of parameter transfer. Image adapted from Poelzl[1]

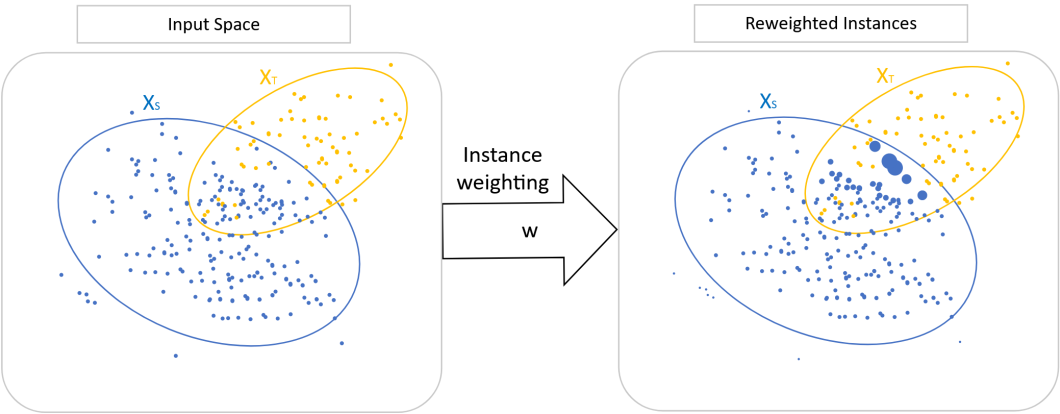

Figure 3. Instance-based Domain Adaptation, adapted from ADAPT[10] and Poelzl[1]

References

1. Poelzl, M. Feasibility of transfer learning for the prediction of particulate matter, DOI: https://dx.doi.org/10.3217/54cvr-72h51 (2023).

2. Pardoe, D. & Stone, P. Boosting for regression transfer. In *Proceedings of the 27th International Conference on International Conference on Machine Learning*, ICML'10, 863–870 (Omnipress, Madison, WI, USA, 2010).

3. Sun, B., Feng, J. & Saenko, K. Return of Frustratingly Easy Domain Adaptation. DOI: 10.48550/ARXIV.1511.05547 (2015). Publisher: arXiv Version Number: 2.

4. Loog, M. Nearest Neighbor-based Importance Weighting (2021). ArXiv:2102.02291 [cs, stat].

5. Sugiyama, M. *et al.* Direct importance estimation for covariate shift adaptation. *Annals Inst. Stat. Math.* **60**, 699–746, DOI: 10.1007/s10463-008-0197-x (2008).

6. Wen, J., Greiner, R. & Schuurmans, D. Correcting covariate shift with the frank-wolfe algorithm. In Yang, Q. & Wooldridge, M. J. (eds.) *Proceedings of the Twenty-Fourth International Joint Conference on Artificial Intelligence, IJCAI 2015, Buenos Aires, Argentina, July 25-31, 2015*, 1010–1016 (AAAI Press, 2015).

7. Zhang, W., Deng, L., Zhang, L. & Wu, D. A Survey on Negative Transfer. *IEEE/CAA J. Autom. Sinica* **10**, 305–329, DOI: 10.1109/JAS.2022.106004 (2023).

8. Pan, S. J. & Yang, Q. A Survey on Transfer Learning. *IEEE Transactions on Knowl. Data Eng.* **22**, 1345–1359, DOI: 10.1109/TKDE.2009.191 (2010).

9. Xiong, P. *et al.* Application of Transfer Learning in Continuous Time Series for Anomaly Detection in Commercial Aircraft Flight Data. In *2018 IEEE International Conference on Smart Cloud (SmartCloud)*, 13–18, DOI: 10.1109/SmartCloud.2018.00011 (IEEE, New York, NY, 2018).

10. de Mathelin, A. *et al.* ADAPT : Awesome Domain Adaptation Python Toolbox (2023). ArXiv:2107.03049 [cs].
